# Big Endothelin-1 as a Predictor of Reverse Remodeling and Prognosis in Dilated Cardiomyopathy

**DOI:** 10.3390/jcm12041363

**Published:** 2023-02-08

**Authors:** Jiayu Feng, Lin Liang, Yuyi Chen, Pengchao Tian, Xuemei Zhao, Boping Huang, Yihang Wu, Jing Wang, Jingyuan Guan, Liyan Huang, Xinqing Li, Yuhui Zhang, Jian Zhang

**Affiliations:** 1 State Key Laboratory of Cardiovascular Disease, Heart Failure Center, National Center for Cardiovascular Diseases, Fuwai Hospital, Chinese Academy of Medical Sciences and Peking Union Medical College, Beijing 100037, China; 2 Key Laboratory of Clinical Research for Cardiovascular Medications, National Health Committee, Beijing 100037, China

**Keywords:** biomarker, dilated cardiomyopathy, reverse remodeling, predictive model

## Abstract

This study aimed to investigate the predictive value of Big endothelin-1(ET-1) for left ventricular reverse remodeling (LVRR) and prognosis in patients with dilated cardiomyopathy (DCM). Patients with DCM and a left ventricular ejection fraction (LVEF) ≤ 50% from 2008 to 2017 were included. LVRR was defined as the LVEF increased by at least 10% or follow-up LVEF increased to at least 50% with a minimum improvement of 5%; meanwhile, the index of left ventricular end-diastolic diameter (LVEDDi) decreased by at least 10% or LVEDDi decreased to ≤33 mm/m^2^. The composite outcome for prognostic analysis consisted of death and heart transplantations. Of the 375 patients included (median age 47 years, 21.1% female), 135 patients (36%) had LVRR after a median of 14 months of treatment. An independent association was found between Big ET-1 at baseline and LVRR in the multivariate model (OR 0.70, 95% CI 0.55–0.89, *p* = 0.003, per log increase). Big ET-1, body mass index, systolic blood pressure, diagnosis of type 2 diabetes mellitus (T2DM) and treatment with ACEI/ARB were significant predictors for LVRR after stepwise selection. Adding Big ET-1 to the model improved the discrimination (∆AUC = 0.037, *p* = 0.042 and reclassification (IDI, 3.29%; *p* = 0.002; NRI, 35%; *p* = 0.002) for identifying patients with LVRR. During a median follow-up of 39 (27–68) months, Big ET-1 was also independently associated with the composite outcome of death and heart transplantations (HR 1.45, 95% CI 1.13–1.85, *p* = 0.003, per log increase). In conclusion, Big ET-1 was an independent predictor for LVRR and had prognostic implications, which might help to improve the risk stratification of patients with DCM.

## 1. Introduction

Dilated cardiomyopathy (DCM) is a heterogeneous disorder defined as dilation and dysfunction of cardiac chambers without ischemic heart disease or loading abnormalities, estimated to affect 1:250 to 1:2500 in the population and cause a 5-year mortality rate of 20% despite therapeutic advancements [1,2]. Left ventricular reverse remodeling (LVRR) is characterized as an increase in LVEF and a decrease in the dimension of the left ventricle, which occurs in 30–50% of patients with DCM in different cohorts [3,4]. In addition to becoming an essential prognostic indicator in DCM, LVRR also has clinical implications for the appropriate use of device therapy, such as cardiac resynchronization therapy (CRT) and implantable cardioverter-defibrillators (ICDs), as well as the timing for referral to left ventricular assist device (LVAD) or transplantation [5,6]. However, predicting LVRR in clinical practice is still challenging and unclear, particularly in patients with DCM.

Big endothelin-1 (Big ET-1) is a 39-amino acid precursor with a much higher concentration and a longer half-life than ET-1 [7]. ET-1 increases collagen production in cardiac fibroblasts, associated with interstitial and vascular fibrosis in cardiac remodeling. ET-1 also has vasoconstriction and inotropic effects on the cardiovascular system [8,9]. Previous studies have shown the association between Big ET-1 and outcome in patients with heart failure (HF), atrial fibrillation (AF), hypertrophic cardiomyopathy (HCM) and left ventricular non-compaction cardiomyopathy (LVNC) [10,11,12,13]. Nevertheless, there is limited evidence regarding Big ET-1’s clinical utility in predicting LVRR for patients with DCM.

Therefore, we aim to investigate the ability of Big ET-1 to predict LVRR beyond clinical parameters, as well as its prognostic implications, to improve the current risk stratification of DCM and promote more accurate screening of patients eligible for advanced therapy.

## 2. Materials and Methods

### 2.1. Study Population

We retrospectively included patients in this study who were first hospitalized for DCM and had an LVEF ≤ 50% at Fuwai Hospital from 2006 to 2017. DCM was defined as myocardial systolic dysfunction and ventricular dilatation that are not explained by any abnormal loading conditions or coronary artery disease (CAD) [1]. We excluded patients (1) who had significant CAD; (2) primary valvular heart disease; (3) congenital heart disease; (4) cancer or autoimmune disease; (5) viral myocarditis; (6) alcoholic cardiomyopathies; (7) left ventricular non-compaction (LVNC); (8) patients with a CRT or CRT-D implantation; and (9) patients missing echocardiography or follow-up data. All participants signed the informed consent form with the approval of the Ethics Committee of Fuwai Hospital (approval numbers 2014-501).

### 2.2. Follow-Up

The participants were re-evaluated and received appropriate medical treatment at Fuwai Hospital as directed by the guideline during follow-up. The follow-up echocardiograms at least 6 months after the baseline examination of patients were searched for in the electronic medical record system. In patients with more than one record, the follow-up echocardiogram was determined based on the examination date closest to 12 months after baseline.

In this study, the definition of LVRR was the presence of both during follow-up: (1) LVEF increased by at least 10% or follow-up LVEF increased to at least 50% with a minimum improvement of 5%, and (2) LVEDDi decreased by at least 10% or LVEDDi decreased to ≤33 mm/m^2^. The composite outcome for prognostic analysis consisted of all-cause death and heart transplantations.

### 2.3. Laboratory Measurement

A vein blood sample was obtained from patients in the morning following admission. Under standard procedures, all biomarkers were tested at the central laboratory. The measurement of N-terminal Pro Brain natriuretic peptide (NT-Pro-BNP) was performed using a commercial enzyme immunoassay (Biomedica, Vienna, Austria or Bio-Tek, Winooski, VT, USA), and the measurement of Big ET-1 was carried out using a commercial sandwich enzyme immunoassay (BI-2008 2H, Biomedica, Vienna, Austria).

### 2.4. Statistical Analysis

We compared the characteristics of patients with or without LVRR using a χ^2^ test for categorical variables and the Mann–Whitney U-test for continuous variables. Comparison of ∆LVEF/LVEDDi between patients with or without LVRR was tested through the interaction in repeated measures using analysis of variance (ANOVA).

We conducted a logistic regression to analyze the relationship between baseline Big ET-1 with other parameters and LVRR. The levels of biomarkers such as Big ET-1, NT-pro-BNP and serum creatine were log2 transformed. Variables that were significantly associated with LVRR (*p* < 0.1) in the univariable analysis were included in the multivariable stepwise regression in backward and forward directions guided by Akaike information criteria (AIC). The association between predictors and LVRR was shown as odds ratio (OR) and a 95% confidence interval (CI). We build the best prediction model of LVRR by including predictors with *p* < 0.05 in the stepwise model. At last, we measured the discrimination, calibration and reclassification of the model adding Big ET-1 in predicting LVRR using the area under curves (AUC), AIC, integrated discrimination improvement (IDI) and net reclassification improvement (NRI), respectively. The discrimination of the predictive model for LVRR was also tested in three-fold 100-time cross-validation. For sensitivity analysis, we fitted a logistic regression model including clinically significant variables and the interval between two echoes to test the consistency of the relationship between Big ET-1 and LVRR.

The Kaplan–Meier curves were plotted to compare the incidence of death or cardiac transplantation in patients with or without LVRR. Cox regressions were conducted to assess the prognostic value of baseline Big ET-1 using stepwise regression with a *p*-value of 0.1 as a significance level for entry and stay. Then, we plotted a time-dependent receiver operating characteristic (ROC) curve at 1, 3 and 5 years to evaluate the accuracy of the Cox model in predicting the prognosis outcome. Hazard ratio (HR) and a 95% confidence interval were reported, and *p* < 0.05 was considered statistically significant. Statistical analysis was performed by the R software 4.1.3.

## 3. Results

### 3.1. Baseline Characteristics

**Figure 1:**
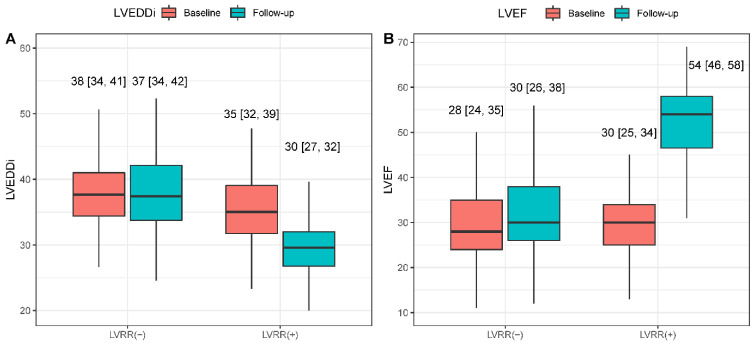
Changes of LVEDDi and LVEF in DCM patients with LVRR and without (**A**,**B**). LVEDDi: left ventricular end-diastolic diameter index; LVEF: left ventricular ejection fraction; LVRR: left ventricular remodeling.

A total of 375 hospitalized patients diagnosed with DCM were included in this study. A summary of baseline characteristics is shown in Table 1, stratified by the presence of LVRR. The median age of included patients was 47 (34–57) years, and 79 (21.1%) patients were female. Compared to patients without LVRR, patients with LVRR had higher baseline blood pressure, heart rate, body mass index (BMI) and cholesterol but lower levels of Big ET-1 and NT-Pro-BNP. Patients with LVRR were more likely to have a history of T2DM and have non-sustained ventricular tachycardia (NSVT) during hospitalization, and have a higher proportion to use angiotensin-converting enzyme inhibitors (ACEI) or angiotensin II receptor blocker (ARB). Regarding baseline echocardiography parameters, patients with LVRR tended to have smaller left atria and ventricular dilation. During a median interval of 14 (12–24) months between baseline and follow-up echocardiogram, 135 patients (36%) had achieved LVRR. A significant reduction in LVEDDi and a significant improvement in LVEF were observed in patients with LVRR compared with patients without LVRR at follow-up (Figure 1, *p* for interaction < 0.001). The baseline characteristics stratified using the median of Big ET-1 are shown in Table S1.

### 3.2. Predictors for LVRR

In the univariable logistic analysis, the baseline Big-ET 1 was associated with the presence of LVRR (crude OR 0.67, 95% CI 0.55–0.83, *p* < 0.001, per log increase). After putting parameters that were significant in univariable regression (*p* < 0.1) in the stepwise regression and selecting predictors by AIC, independent baseline predictors of LVRR included Big ET-1 (OR 0.70, 95% CI 0.55–0.89, *p* = 0.003, per log increase), BMI (OR 1.07, 95% CI 1.01–1.14, *p* = 0.026), SBP (OR 1.02, 95% CI 1.01–1.04, *p* = 0.035), diagnosis of T2DM (OR 0.30, 95% CI 0.14–0.65, *p* = 0.002) and the treatment with ACEI/ARB (OR 2.30, 95% CI 1.02–5.21, *p* = 0.045). The association between variables and LVRR at univariable and multivariable logistic regression is shown in Table 2. For sensitivity analysis, we fitted a regression model including all clinically significant variables and the interval between two echoes, and the Big ET-1 still showed an independent association with LVRR (Table S2).

### 3.3. Predictive Value of Adding Big ET-1 to the Model for LVRR

**Figure 2:**
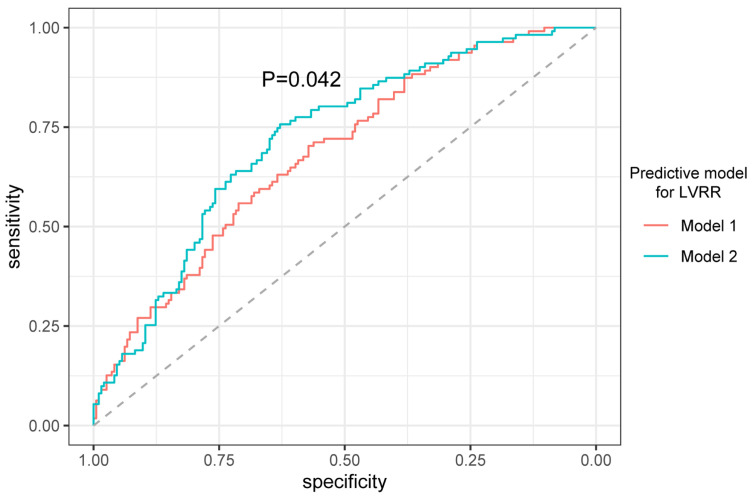
The receiver-operating characteristic curves (ROC) of the model before and after adding Big ET-1 in predicting LVRR. The basic prediction model (model 1) constructed using stepwise regression [including BMI, SBP, diagnosis of T2DM and the treatment with ACEI/ARB]. Model 2 included parameters in model 1 adding log 2-transformed Big ET-1.

The performance of the predictive model after adding Big ET-1 is shown in Table 3. In terms of discrimination, adding Big ET-1 improved the AUC from 0.684 to 0.721 (*p* = 0.042, Figure 2), and improved the mean AUC from 0.653 to 0.682 after cross-validation. The AIC decreased after adding Big ET-1 to the basic model (model 2: 367.25 vs. model 1: 375.94), which showed that including Big ET-1 improved the calibration of the predictive model. Moreover, the model including Big ET-1 (model 2) significantly enhanced the reclassification for patients with LVRR compared with model 1 (IDI, 3.29%; *p* = 0.002; NRI, 35%; *p* = 0.002).

### 3.4. The Outcome of Patients with LVRR and Prognostic Role of Big ET-1

**Figure 3:**
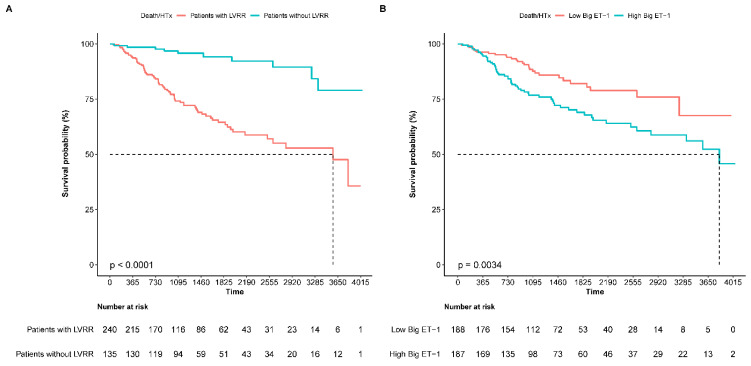
Comparison of the outcome between patients with LVRR (+) and LVRR (−) (**A**), and between patients with high Big ET-1 and low Big ET-1 (**B**). The high Big ET-1 was defined as the Big ET-1 above the median (0.72 pmol/L). Death/HTx: all-cause death or heart transplantations.

During a median follow-up of 39 (27–68) months, 66 patients died (17.6%) and 19 patients underwent heart transplants (5.1%). DCM patients with LVRR had a better composite outcome than those without LVRR (Figure 3A). After adjusting for confounders, patients with LVRR still had a lower risk of death or heart transplantations (adjusted HR 0.27, 95% CI 0.14–0.52, *p* < 0.001). In terms of the relationship between Big ET-1 and long-term prognosis, when the Big ET-1 was dichotomized by the median, the high Big ET-1 group (Big ET-1 > 0.72 pmol/L) was more likely to reach the composite endpoint than the low Big ET-1 group (Figure 3B). In the multivariable regression, baseline Big ET-1 was also independently associated with the outcome as a categorical (adjusted HR 1.94, 95% CI 1.18–3.18, *p* = 0.009) or a continuous variable (adjusted HR 1.45, 95% CI 1.13–1.85, *p* = 0.003, per log increase, Table 4). The restricted cubic splines (using four knots) of the relationship between log Big ET-1 and outcome are shown in Figure S1. We screened five significant variables to build a predictive model for all-cause death and heart transplantation through COX stepwise regression, including age, SBP, LVEDD, log NT-Pro-BNP, use of ACEI/ARB and log Big ET-1. Through time ROC analysis, we found that the prognosis prediction model including Big ET-1 can reach AUCs of 0.84, 0.77 and 0.78 for 1-, 3- and 5-year event-free survival (Figure S2).

## 4. Discussion

We studied the role of Big ET-1 in predicting LVRR and prognosis in a cohort of DCM patients. We demonstrated that patients with LVRR showed a lower risk of adverse events. Importantly, baseline Big ET-1 proved to be a significant predictor for LVRR as well as the clinical outcome. The predictive model with Big ET-1 for LVRR can improve the current risk stratification of DCM, strengthen the monitoring of high-risk patients and promote more accurate screening of patients eligible for advanced therapy.

The reverse remodeling process involves normalizing chamber geometry and function in the failing myocardium. Previous studies have shown that despite reverse remodeling, the myocardium exhibits abnormal gene expression, metabolism pathways, and extracellular matrix structure, indicating that remission is not the same as recovery in myocardial function [14,15]. However, several research results suggest that the earlier the therapy of reverse remodeling is applied, the more apparent cardiac structure and function recovery will be achieved [14,16,17]. As shown in this study, LVRR predicts a better outcome, which is also confirmed by a meta-analysis reporting a relationship between short-term changes in LV structure and function with effects on survival [18]. Therefore, the current challenge is the early stratification to identify patients with a lower probability of LVRR and the most appropriate therapy.

Previous studies have confirmed that circulating biomarkers such as NT-Pro-BNP, high-sensitivity troponin, soluble ST2, and galectin-3 may help to identify LVRR [19,20,21]. In this study, our results showed that Big ET-1, a precursor of ET-1, also has a predictive value for LVRR. The potential mechanism of Big ET-1 in predicting LVRR may be related to the biological effects of ET receptor activation on cardiomyocytes or fibroblasts. The ET(A) receptor activates several pathways in HF and cardiac remodeling, including vasoconstriction, inotropy, hypertrophy and fibrosis [8,9]. Additionally, a variety of vasoactive agents (such as NE, angiotensin II, and thrombin) and cytokines (such as TGF-β, TNF, and IL-1) can enhance ET release in vitro [22]. Therefore, the lower level of Big ET-1 may indicate a less severe neuroendocrine activation or cardiac overload, which is more likely to reach myocardial remission after drug treatment. A recent basic study demonstrated the endothelial Forkhead Box transcription factor P1 (EC-Foxp-1) gain of function in protecting from cardiac remodeling and improving cardiac dysfunction by directly downregulating the Transforming Growth Factor-β1 (TGF-β1) gene, which promotes the ET-1 expression [23]. This study supports the relationship between ET-1 and LVRR from the mechanism perspective. In addition, it suggests that a novel therapy targeting the TGF-β1–ET-1 pathway might help relieve or reverse the remodeling of the heart.

Several reports have shown that higher Big ET-1 levels correlate with worse prognosis in patients with HF [24,25]. Our study has confirmed this conclusion in patients with HF caused by DCM, and the prognosis prediction models including Big ET-1 showed high accuracy. Previous studies have measured Big ET-1 serially and found that the decline of Big-ET-1 during follow-up after 1 month was associated with less adverse events in patients with ischemic HF [26], demonstrating that the monitoring of Big ET-1 at follow-up may provide additional prognostic value. Conversely, results from the Multi-Ethnic Study of Atherosclerosis Angiogenesis (MEAS) Sub-Study suggested that higher ET-1 levels measured in patients without cardiovascular disease may be associated with a lower risk for incident HF or cardiovascular death [27]. This finding agrees with previous results in pre-clinical animal models that suggest even a modest (~35%) decrease in endothelin-1 gene (Edn1) expression is sufficient to cause cardiac dysfunction [28]. The increase in ET-1 is related to the severity and prognosis of patients with HF, but may have cardioprotective effects on normal hearts.

In addition to the Big ET-1, BMI, SBP, diagnosis of T2DM and treatment with ACEI/ARB were also identified as predictors for LVRR. T2DM is proven to be associated with worse LV systolic and diastolic function in DCM patients, and hemoglobin A1C (HbA1c) is related to reduced LV myocardial strain [29]. Our results showed that patients without LVRR had a higher proportion of T2DM and a higher level of HbA1c, despite no significant difference in fast blood glucose (FBG) between patients with LVRR present or absent. Our findings indicated the importance of diabetes management and HbA1c monitoring for patients with DCM. A meta-analysis on sodium–glucose cotransporter-2 (SGLT2) inhibitors and LVRR included 13 trials and a total of 1251 patients with T2DM and/or HF, which found that SGLT2 inhibitors significantly improve LVEF, LV mass index, LVESV index and E-wave deceleration time during follow-up [30]. However, the current study included patients hospitalized from 2006 to 2017; no patients in this study were administrated SGLT2 inhibitors during this period. Nowadays, SGLT2i is being popularized and applied in patients with HF according to new guidelines; further research should analyze the impact of SGLT2i use on LVRR in DCM patients with or without T2DM in real-world situations. Patients with LVRR had a higher prescription of ACEI/ARB, which might be related to the higher blood pressure and better tolerance for drugs of these patients. Therefore, for patients with DCM, avoiding hypotension and titration of standard therapies plays a vital role in the cardiac reverse remodeling.

However, this study had several limitations. First, this study was retrospective, and the sample size of patients who had follow-up echocardiograms was relatively small; therefore, selection bias and some potential confounders might not be fully adjusted. Second, the proportion of patients with dynamic monitoring of Big ET-1 during follow-up is low; thus, this study could not analyze the association between the dynamic change and LVRR. Lastly, the prediction model established in this study has not been verified by an external cohort; thus, the generalization of predictive models should be cautious and requires further external validation.

## 5. Conclusions

An increased level of serum Big ET-1 was independently associated with LVRR and prognosis in a cohort of Chinese patients with DCM. The predictive model including Big ET-1, BMI, SBP, diagnosis of T2DM and the treatment with ACEI/ARB showed a predictive value for LVRR. The establishment of a predictive model including Big ET-1 helps to improve the risk stratification of patients with DCM and select high-risk patients with a lower probability of LVRR to receive stricter management or device therapy.

## Figures and Tables

**Table 1:** Baseline characteristics for DCM patients with LVRR absent and LVRR present.

	Overall	LVRR Absent	LVRR Present	*p*-Value
N	375	240	135	
**Clinical characteristics**				
Age(years)	47 [34, 57]	48 [35, 58]	44 [32, 55]	0.073
Female (%)	79 (21.1)	52 (21.7)	27 (20.0)	0.804
Heart rate (b.p.m)	84 [72, 96]	82 [70, 94]	85 [77, 98]	0.023
SBP (mmHg)	113 [102, 124]	110 [100, 121]	116 [105, 130]	0.001
DBP (mmHg)	72 [66, 80]	70 [65, 80]	76 [70, 85]	<0.001
BMI (kg/m^2^)	24.8 [22.1, 28.1]	24.5 [21.7, 27.3]	26.0 [23.2, 29.2]	0.002
T2DM (%)	66 (17.6)	51 (21.2)	15 (11.1)	0.020
Hypertension (%)	124 (33.1)	64 (26.7)	60 (44.4)	0.001
NYHA Class III/IV (%)	297 (79.2)	196 (81.7)	101 (74.8)	0.151
Smoking (%)	123 (49.4)	81 (51.6)	42 (45.7)	0.439
Length of stay (days)	10 [8, 13]	11 [8, 14]	10 [8, 13]	0.153
**Electrocardiography**				
QRS duration (ms)	107 [95, 126]	110 [98, 128]	101 [92, 115.2]	0.001
PR interval (ms)	174 [160, 192]	174 [158, 192]	176 [161, 190]	0.913
QTc interval (ms)	457 [430, 486]	457 [431, 486]	456 [427, 478]	0.298
AF (%)	87 (23.2)	60 (25.0)	27 (20.0)	0.330
LBBB (%)	36 (9.6)	26 (10.8)	10 (7.4)	0.369
NSVT (%)	88 (23.5)	66 (27.5)	22 (16.3)	0.020
**Laboratory Test**				
Hemoglobin (g/L)	150.0 [137.2, 162.0]	148.0 [137.0, 159.0]	153.0 [139.5, 167.0]	0.010
WBC (10^9^/L)	7.4 [6.2, 8.9]	7.2 [6.2, 8.9]	7.6 [6.3, 9.1]	0.330
K (mmol/L)	4.0 [3.7, 4.2]	4.0 [3.7, 4.2]	4.0 [3.7, 4.2]	0.996
Na (mmol/L)	138.0 [135.4, 140.0]	137.3 [134.9, 139.9]	139.0 [136.4, 140.9]	0.001
FBG (mmol/L)	5.1 [4.6, 5.7]	5.1 [4.6, 5.8]	5.0 [4.6, 5.5]	0.299
Hemoglobin A_1C_ (%)	6.1 [5.6, 6.7]	6.2 [5.7, 6.9]	6.0 [5.6, 6.5]	0.039
LDL-C (mmol/L)	2.7 [2.1, 3.4]	2.6 [2.0, 3.3]	2.9 [2.2, 3.4]	0.028
Scr (umol/L)	89.4 [75.5, 106.2]	89.4 [75.6, 103.8]	89.4 [75.5, 108.0]	0.974
Big ET-1 (pmol/L)	0.72 [0.41, 1.02]	0.77 [0.52, 1.08]	0.61 [0.30, 0.94]	<0.001
NT-Pro BNP (pg/mL)	1922.9 [897.4, 4021.5]	2041.9 [1078.5, 4297.5]	1570.0 [745.3, 3265.0]	0.009
**Echocardiography**				
LAD (mm)	45 [41, 50]	46 [41, 51]	44 [40, 48]	0.002
LVEDD (mm)	68 [63, 74]	69 [63, 75]	66 [62, 72]	0.021
LVEF (%)	29 [24, 35]	28 [24, 35]	30 [25, 34]	0.236
RVD (mm)	25 [22, 28]	25 [22, 29]	24 [22, 28]	0.199
**Therapy**				
Digoxin (%)	324 (86.4)	208 (86.7)	116 (85.9)	0.965
ACEI/ARB (%)	305 (81.3)	184 (76.7)	121 (89.6)	0.003
β-blocker (%)	366 (97.6)	232 (96.7)	134 (99.3)	0.221
MRA (%)	364 (97.1)	231 (96.2)	133 (98.5)	0.352
Diuretics (%)	307 (81.9)	197 (82.1)	110 (81.5)	0.996

Values are shown as median [interquartile range] or as frequencies [percentage]; DCM: dilated cardiomyopathy; LVSD: left ventricular systolic dysfunction; SBP: systolic blood pressure; DBP: diastolic blood pressure; BMI: body mass index; T2DM: Type 2 diabetes mellitus; NYHA, New York Heart Association; AF: atrial fibrillation; LBBB: left bundle branch block; NSVT: Non-sustained ventricular tachycardia; WBC: white blood cell; LDL-C: low density cholesterol; Scr: serum creatine; Big ET-1: Big endothelin-1; NT-Pro BNP: N-terminal Pro Brain natriuretic peptide; LAD: left atrial diameter; LVEDD: left ventricular end-diastolic diameter; LVEF: left ventricular ejection fraction; RVD: right ventricular diameter; ACEI: Angiotensin converting enzyme inhibitor; ARB: Angiotensin receptor blocker; MRA: mineralocorticoid receptor antagonists.

**Table 2:** The association between variables and LVRR at univariable and multivariable logistic regression.

	Univariable Logistic Regression		Multivariable Logistic Regression	
Characteristics	Crude OR (95%CI)	*p*-Value	Adjusted OR (95%CI)	*p*-Value
Age	0.99 (0.97–1.01)	0.098		
Gender	0.9 (0.54–1.52)	0.704		
**BMI**	**1.07 (1.03–1.12)**	**0.003**	**1.07 (1.01–1.14)**	**0.026**
NYHA III/IV	0.67 (0.4–1.11)	0.118		
**SBP**	**1.03 (1.01–1.04)**	**<0.001**	**1.02 (1.01–1.04)**	**0.035**
**T2DM**	**0.46 (0.25–0.86)**	**0.015**	**0.30 (0.14–0.65)**	**0.002**
AF	0.75 (0.45–1.25)	0.272		
QRS duration	0.90 (0.82–0.98)	0.015	0.92 (0.84–1.02)	0.124
LBBB	0.66 (0.31–1.41)	0.282		
LVEF	1.01 (0.99–1.04)	0.325		
LVEDD	0.97 (0.95–1.01)	0.024	0.98 (0.95–1.01)	0.167
RVD	0.97 (0.93–1.01)	0.147		
Hemoglobin	1.02 (1.01–1.03)	0.009		
**log Big ET-1**	**0.67 (0.55–0.83)**	**<0.001**	**0.70 (0.55–0.89)**	**0.003**
log NT-Pro-BNP	0.84 (0.74–0.96)	0.012		
log Scr	0.76 (0.45–1.29)	0.304		
**ACEI/ARB**	**2.63 (1.40–4.93)**	**0.003**	**2.30 (1.02–5.21)**	**0.045**
β-blocker	4.62 (0.57–37.35)	0.151		
MRA	2.59 (0.55–12.17)	0.228		

Variables that were significantly associated with LVRR (*p* < 0.1) in the univariable analysis were included in the multivariable stepwise regression in backward and forward directions guided by AIC. Independent predictors for LVRR (*p* < 0.05 in multivariable model) are shown in bold. The levels of biomarkers such as Big ET-1, NT-pro-BNP and serum creatine were log2 transformed. SBP: systolic blood pressure; BMI: body mass index; T2DM: Type 2 diabetes mellitus; NYHA, New York Heart Association; AF: atrial fibrillation; LBBB: left bundle branch block; LVEDD: left ventricular end-diastolic diameter; LVEF: left ventricular ejection fraction; RVD: right ventricular diameter; Scr: serum creatine; Big ET-1: Big endothelin-1; NT-Pro BNP: N-terminal Pro Brain natriuretic peptide; ACEI: Angiotensin converting enzyme inhibitor; ARB: Angiotensin receptor blocker; MRA: mineralocorticoid receptor antagonists.

**Table 3:** Discrimination and reclassification of adding Big ET-1 to the model for LVRR.

	Model 1 (Basic Model)	Model 2 (Basic Model + Big ET-1)	*p*-Value
**AUC**	0.684 (0.624–0.744)	0.721 (0.663–0.779)	0.042
**IDI**	reference	0.033 (0.012,0.054)	0.002
**cNRI**	reference	0.354 (0.1253–0.583)	0.002

The basic prediction model constructed using stepwise regression [including BMI, SBP, diagnosis of T2DM and the treatment with ACEI/ARB]. cNRI and IDI of the additional value of Big ET-1 was calculated at 5 years of follow-up. The level of Big ET-1 was log2 transformed.

**Table 4:** Independent predictors for the composite of all-cause death and heart transplantations.

	Crude HR (95%CI)	Crude *p* Value	Adjusted HR (95% CI)	Adjusted *p* Value
Age	1.01 (1.00,1.03)	0.066	1.03 (1.01,1.04)	<0.001
SBP	0.96 (0.95,0.98)	<0.001	0.97 (0.95,0.99)	<0.001
LVEDD	1.06 (1.04,1.09)	<0.001	1.06 (1.03,1.09)	<0.001
log Big ET-1	1.49 (1.20,1.85)	<0.001	1.45 (1.13,1.85)	0.003
log NT-Pro-BNP	1.55 (1.33,1.80)	<0.001	1.29 (1.08,1.54)	0.005
Use of ACEI/ARB	0.40 (0.25,0.64)	<0.001	0.52 (0.32,0.86)	0.011

Cox regressions were conducted using stepwise regression with a *p*-value of 0.1 as a significance level for entry and stay. SBP: systolic blood pressure; BMI: body mass index; LVEDD: left ventricular end-diastolic diameter; Big ET-1: Big endothelin-1; NT-Pro BNP: N-terminal Pro Brain natriuretic peptide; ACEI: Angiotensin converting enzyme inhibitor; ARB: Angiotensin receptor blocker.

## Data Availability

Not applicable.

## Supplementary Materials

The following supporting information can be downloaded at: https://www.mdpi.com/article/10.3390/jcm12041363/s1, Table S1: Baseline characteristics for DCM patients with Big ET-1 ≤median vs. >median. Table S2: Sensitivity analysis for logistic regression model including clinically significant variables and the interval between two echoes. Figure S1: The restricted cubic splines of the relationship between log Big ET-1 and outcome. Figure S2: Time ROC plot for the model including Big ET-1 in predicting the composite of all-cause death and heart transplantations.
