# Supplementary material for: Big Endothelin-1 as a Predictor of Reverse Remodeling and Prognosis in Dilated Cardiomyopathy
Source: J Clin Med. 2023 Feb 8;12(4):1363. doi: 10.3390/jcm12041363 (PMC9967115; doi:10.3390/jcm12041363)
Supplement: Supplementary file 1 [file jcm-12-01363-s001.zip › jcm-2177065-supplementary.pdf]

Table S1 Baseline characteristics for DCM patients with Big ET-1≤ median vs. >median.

	Overall	Big ET-1≤median	Big ET-1>median	P-Value
N	375	188	187	
**Clinical characteristics**				
Age(years)	47 [34, 57]	48 [34, 57]	46 [34, 57]	0.275
Female (%)	79 (21.1)	41 (21.8)	38 (20.3)	0.821
Heart rate (b.p.m)	84 [72, 96]	80 [71, 95]	85 [75, 96]	0.11
SBP (mmHg)	113 [102, 124]	116 [105, 127]	110 [100, 122]	0.011
DBP (mmHg)	72 [66, 80]	73.5 [65, 80]	71 [66, 80]	0.981
BMI (kg/m2)	24.8 [22.1, 28.1]	24.8 [22.1, 27.8]	24.8 [22.3, 28.2]	0.781
T2DM (%)	66 (17.6)	23 (12.2)	43 (23.0)	0.009
Hypertension (%)	124 (33.1)	67 (35.6)	57 (30.5)	0.341
NYHA Class III/IV (%)	297 (79.2)	134 (71.3)	163 (87.2)	<0.001
Smoking (%)	123 (49.4)	59 (50.4)	64 (48.5)	0.858
Length of stay (days)	10 [8, 13]	10 [8, 13]	11 [8, 14]	0.011
**Electrocardiography**				
QRS duration (ms)	107 [95, 126]	104 [94, 118.5]	110 [96, 130]	0.131
PR interval (ms)	174 [160, 192]	174 [158.9, 189.2]	176 [160, 197.6]	0.45
QTc interval (ms)	457 [430, 486]	454 [428, 484]	461 [433, 488]	0.133
AF (%)	87 (23.2)	42 (22.3)	45 (24.1)	0.785
LBBB (%)	36 (9.6)	19 (10.1)	17 (9.1)	0.874
NSVT (%)	88 (23.5)	47 (25.0)	41 (21.9)	0.561
**Laboratory Test**				
Haemoglobin (g/L)	150.0 [137.2, 162.0]	150.0 [139.0, 164.0]	149.0 [135.0, 159.5]	0.106
WBC (10^9/L)	7.4 [6.2, 8.9]	7.1 [6.0, 8.8]	7.6 [6.4, 9.1]	0.044
K (mmol/L)	4.0 [3.7, 4.2]	3.9 [3.7, 4.2]	4.0 [3.7, 4.3]	0.833
Na (mmol/L)	138.0 [135.4, 140.0]	138.8 [136.0, 140.9]	137.3 [134.9, 139.6]	0.001
FBG (mmol/L)	5.1 [4.6, 5.7]	5.0 [4.6, 5.7]	5.1 [4.6, 5.9]	0.537
Hemoglobin A1C (%)	6.1 [5.6, 6.7]	6.0 [5.6, 6.4]	6.3 [5.7, 6.9]	0.002
LDL-C (mmol/L)	2.7 [2.1, 3.4]	2.8 [2.3, 3.3]	2.5 [2.0, 3.4]	0.038
Scr (umol/L)	89.4 [75.5, 106.2]	87.5 [73.2, 108.0]	91.2 [78.3, 104.3]	0.264
Big ET-1 (pmol/L)	0.72 [0.41, 1.02]	0.41[0.24, 0.58]	1.02 [0.85, 1.38]	<0.001
NT-Pro BNP (pg/ml)	1922.9 [897.4, 4021.5]	1440.9 [677.2, 2693.2]	2355.9 [1185.0, 5110.0]	<0.001
**Echocardiography**				
LAD (mm)	45 [41, 50]	45 [40, 49]	46 [42, 52]	0.003
LVEDD (mm)	68 [63, 74]	68 [62, 73]	68 [63, 75]	0.559
LVEF (%)	29 [24, 35]	30 [25, 35]	28 [23, 33]	0.057
RVD (mm)	25 [22, 28]	24 [22, 26]	26 [23, 29]	<0.001
**Therapy**				
Digoxin (%)	324 (86.4)	165 (87.8)	159 (85.0)	0.533
ACEI/ARB (%)	305 (81.3)	152 (80.9)	153 (81.8)	0.914

β-blocker (%)	366 (97.6)	186 (98.9)	180 (96.3)	0.175
MRA (%)	364 (97.1)	185 (98.4)	179 (95.7)	0.218
Diuretics (%)	307 (81.9)	153 (81.4)	154 (82.4)	0.913

Values are shown as median [interquartile range] or as frequencies [percentage];

DCM: dilated cardiomyopathy; LVSD: left ventricular systolic dysfunction; SBP: systolic blood pressure; DBP: diastolic blood pressure; BMI: body mass index; T2DM: Type 2 diabetes mellitus; NYHA, New York Heart Association; AF: atrial fibrillation; LBBB: left bundle branch block; NSVT: Non-sustained ventricular tachycardia; WBC: white blood cell; LDL-C: low density cholesterol; Scr: serum creatine; Big ET-1: Big Endothelin-1; NT-Pro BNP: N-terminal Pro Brain natriuretic peptide; LAD: left atrial diameter; LVEDD: left ventricular end-diastolic diameter; LVEF: left ventricular ejection fraction; RVD: right ventricular diameter; ACEI: Angiotensin converting enzyme inhibitor; ARB: Angiotensin receptor blocker; MRA: mineralocorticoid receptor antagonists

Table S2 Sensitivity analysis for logistic regression model including clinically significant variables and the interval between two echoes

	crude OR (95%CI)	crude P value	adjusted OR (95%CI)	adjusted P value
log Big ET-1	0.67 (0.55,0.83)	0.001	0.75 (0.57,0.99)	0.045
Age	0.98 (0.97,0.99)	0.029	0.99 (0.97,1.01)	0.37
Gender	1.01 (0.56,1.82)	0.979	0.97 (0.49,1.92)	0.922
BMI	1.06 (1.01,1.12)	0.018	1.06 (0.99,1.13)	0.108
NYHA III/IV	0.69 (0.39,1.24)	0.218	0.90 (0.47,1.73)	0.748
SBP	1.02 (1.01,1.04)	0.005	1.02 (1.01,1.04)	0.03
QRS duration	0.92 (0.83,1.01)	0.069	0.94 (0.84,1.04)	0.233
T2DM	0.43 (0.21,0.86)	0.017	0.37 (0.17,0.84)	0.017
LVEF	1.01 (0.98,1.05)	0.441	0.99 (0.95,1.03)	0.643
LVEDD	0.98 (0.95,1.01)	0.14	0.98 (0.94,1.01)	0.188
RVD	0.96 (0.92,1.01)	0.133	0.99 (0.93,1.05)	0.684
log NT-Pro-BNP	0.87 (0.76,1.01)	0.061	1.03 (0.85,1.24)	0.784
ACEI/ARB	3.45 (1.48,8.08)	0.004	2.36 (0.95,5.88)	0.066
interval between two echoes	0.98 (0.96,0.99)	0.011	0.98 (0.96,0.99)	0.02

SBP: systolic blood pressure; BMI: body mass index; T2DM: Type 2 diabetes mellitus; NYHA, New York Heart Association; LVEDD: left ventricular end-diastolic diameter; LVEF: left ventricular ejection fraction; RVD: right ventricular diameter; NT-Pro BNP: N-terminal Pro Brain natriuretic peptide; ACEI: Angiotensin converting enzyme inhibitor; ARB: Angiotensin receptor blocker

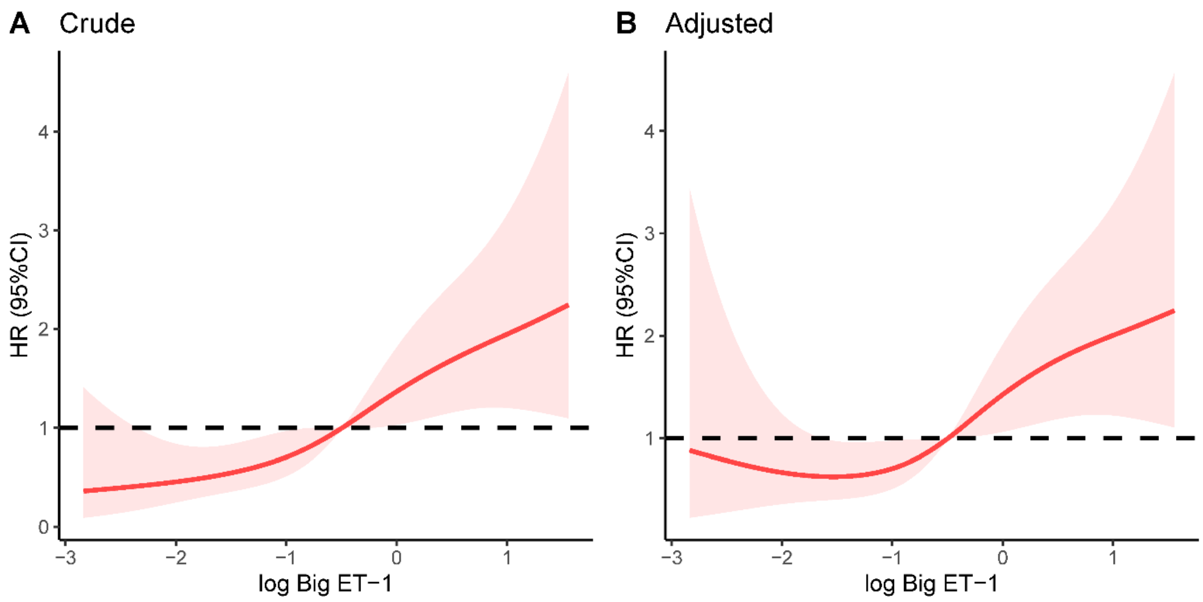

Figure S1 The restricted cubic splines of the relationship between log Big ET-1 and outcome

The multivariable COX regression model included age, SBP, LVEDD, log Big ET-1, log NT-Pro-BNP and use of ACEI/ARB

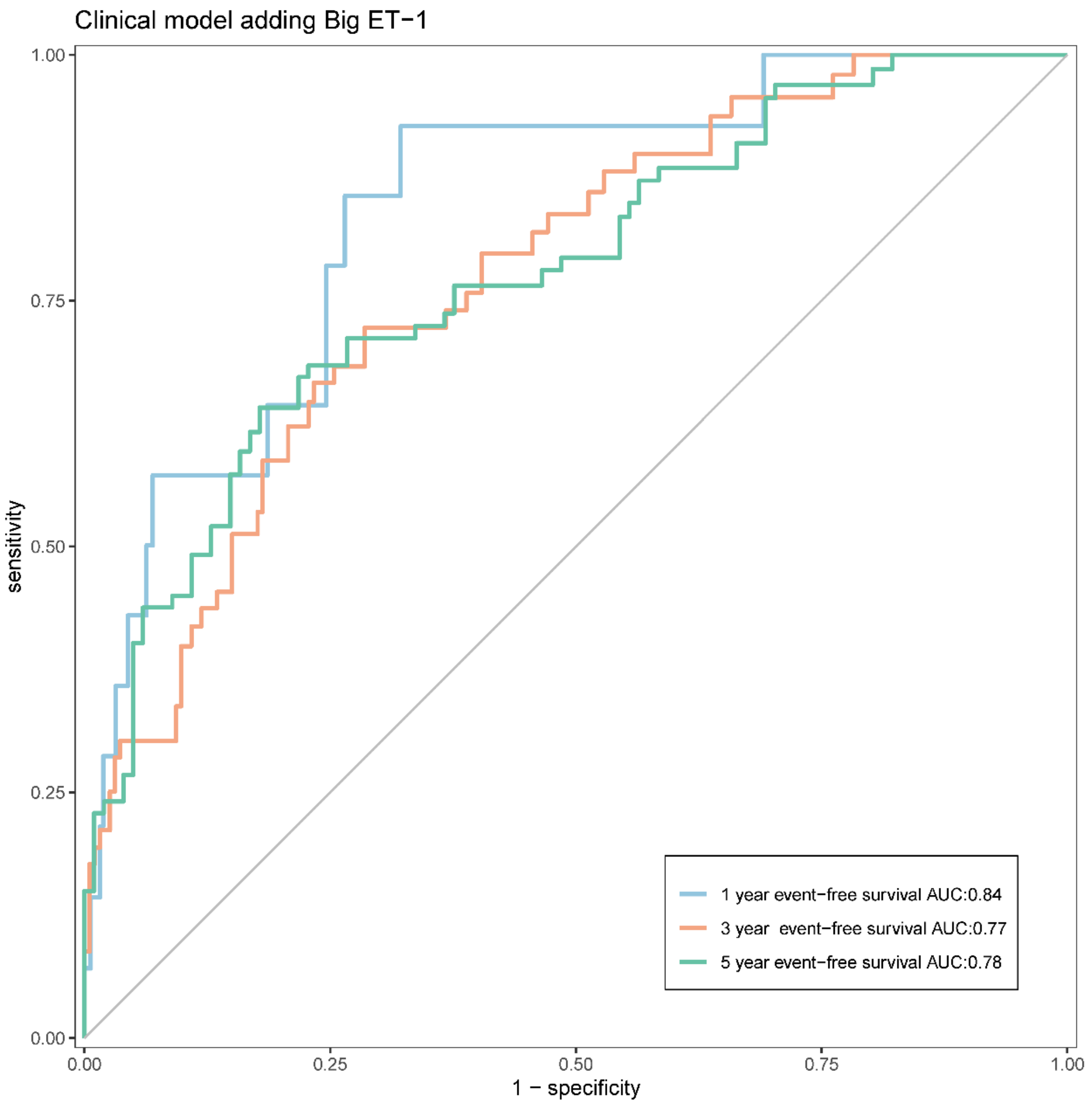

Figure S2 Time ROC plot for the model including Big ET-1 in predicting the composite of all-cause death and heart transplantations

The predictive model for the outcome included age, SBP, LVEDD, log Big ET-1, log NT-Pro-BNP and use of ACEI/ARB. Through Through time ROC analysis, we found that the prognosis prediction models including Big ET-1 can reach AUCs of 0.84, 0.77 and 0.78 for 1-, 3- and 5-years event-free survival
